# State-Degradation-Oriented Fault Diagnosis for High-Speed Train Running Gears System

**DOI:** 10.3390/s20041017

**Published:** 2020-02-13

**Authors:** Chao Cheng, Weijun Wang, Hao Luo, Bangcheng Zhang, Guoli Cheng, Wanxiu Teng

**Affiliations:** 1 School of Computer Science and Engineering, Changchun University of Technology, Changchun 130012, China; chengchao@mail.tsinghua.edu.cn (C.C.); jinwei312@163.com (W.W.); 2 CRRC Changchun Railway Vehicles Co., Ltd., Changchun 130062, China; chengguoli@cccar.com.cn (G.C.); tengwanxiu@cccar.com.cn (W.T.); 3 Department of Automation, Tsinghua University, Beijing 100084, China; 4 Academy of Astronautics, Harbin Institute of Technology, Harbin 150001, China; 5 School of Mechatronic Engineering, Changchun University of Technology, Changchun 130012, China; zhangbangcheng@ccut.edu.cn

**Keywords:** fault diagnosis, distributed state estimation, state degradation

## Abstract

As one of the critical components of high-speed trains, the running gears system directly affects the operation performance of the train. This paper proposes a state-degradation-oriented method for fault diagnosis of an actual running gears system based on the Wiener state degradation process and multi-sensor filtering. First of all, for the given measurements of the high-speed train, this paper considers the information acquisition and transfer characteristics of composite sensors, which establish a distributed topology for axle box bearing. Secondly, a distributed filtering is built based on the bilinear system model, and the gain parameters of the filter are designed to minimize the mean square error. For a better presentation of the degradation characteristics in actual operation, this paper constructs an improved nonlinear model. Finally, threshold is determined based on the Chebyshev’s inequality for a reliable fault diagnosis. Open datasets of rotating machinery bearings and the real measurements are utilized in the case studies to demonstrate the effectiveness of the proposed method. Results obtained in this paper are consistent with the actual situation, which validate the proposed methods.

## 1. Introduction

Nowadays, railway construction and operation are high-speed, which considerably affects the sustainability and the rapid progress of the national economy. The safety and reliability of railway operation have drawn more and more attention. As a core system that directly affects the smooth running of high-speed trains, the running gears system is a key component of the train in providing dynamics and traction performance and has the functions of buffering, vibration isolation, generating power and supporting the vehicle body. As a matter of fact, the running gears system is a complex system that is coupled with many components. Among those components, the corrosion, shedding and degradation of one component may easily spread the local fault and propagate into a major fault in the system level, which may cause unexpected losses. It is very important to analyze system performance degradation [1,2] and prevent fault propagation. Therefore, the timely diagnosis of a running gears system plays a key role in ensuring the safe operation of trains.

In the past few decades, many work has been done on fault diagnosis technologies in different aspects. Traditionally, the fault diagnosis methods for high-speed train systems mainly consist of two factors: The model-based one and the signal-based one [3]. The signal-based fault diagnosis methods analyze the measurement data for fault diagnosis without knowing/identifying the system model. The common signal-based methods include multivariate statistical analysis [4,5], machine learning methods [6,7], signal processing methods [8], information fusion methods [9,10], etc. The above methods have high requirements on data volume and type. The measurements of the running gears system of high-speed trains are of high sampling frequency and long measurement cycle. Once the running gears system of the high-speed train is damaged, it will be repaired in a timely manner and regularly maintained. However, the adequate failure data for analysis are very rare. The most important step of the signal-based fault diagnosis method is the accurate extraction of fault characteristics [11]. If the measurements with inconspicuous fault characteristics are used for the learning, accurate feature extraction normally cannot be guaranteed, and the result of fault diagnosis will be directly affected. In contrast, model-based fault diagnosis methods, which are more sensitive to uncertainty/fault, have lower requirements for data quantity and data type. They are more suitable for a reliable fault diagnosis of high-speed train running gears systems.

Among the model-based fault diagnosis methods, a class of improved methods based on the Kalman filter is used to deal with the influence of system/measurement noise. Yan et al. [12] introduced the extended Kalman filter model and the improved support vector machine model into the online classification framework, where the nonlinearity is approximated by the first-order Taylor expansion and the online fault detection of the ventilation and air-conditioning system is later achieved. Ning et al. [13] proposed an enhanced robust Kalman filtering method based on the optimal radial basis function neural network, which reduces the impact of state errors and observation errors on state estimation. Wang et al. [14] reduced the influence of estimated noise of on-board fault diagnosis and fault-tolerant control through the Kalman filter and improved the robustness of fault diagnosis. Finally, the method is verified by an onboard prototype control system. Akai et al. [15] used the particle filter method to realize the state estimation of a target robot in order to realize system filtering under the influence of noise. Wu et al. [16] proposed a fault diagnosis algorithm for early failures based on the generalized estimation. Yin et al. [17] applied a state resonance algorithm-based stochastic resonance method to high-speed train traction systems to achieve optimal extraction and frequency recovery. Cadini et al. [18] used a particle filter to estimate the length of cracks to diagnose the early faults of aeronautical structure cracks. For state estimation with unknown inputs, some researchers use unknown input residual generators and estimation filters to achieve fault diagnosis [19]. Scholars also consider the decomposition of external disturbances and combine unknown input observers to complete composite fault diagnosis [20]. For nonlinear systems, He et al. [21] applied rank particle filtering to diagnosis the fault of underwater vehicles. In recent years, to improve the accuracy of state estimation and reduce the false alarm rate, scholars have carefully studied the measurement noise and unknown disturbance. Zhou et al. [22] applied an improved odorless Kalman filter and a radial basis function based on an adaptive noise factor to the pumping unit for fault diagnosis. Liu et al. [23] used an adaptive controller to process unknown system parameters and disturbances to achieve target state tracking. Gan et al. [24] used an adaptive factor to improve the accuracy of the unscented Kalman filter algorithm, thereby improving the accuracy of fault diagnosis of analog circuits. However, most of the existing methods are based on state estimation and improvement of a single node of the monitoring system. Such methods mostly rely on the reliability of information collection nodes. For an actual high-speed train information collection system, the measurements obtained in different locations of the same monitoring area contain different information, which will indirectly affect the consistency of information collection and reduce the accuracy of fault diagnosis. With the development of a multi-sensor system, distributed estimation has been studied and applied in different scenarios. Jing et al. [25] realized multi-sensor data fusion of the planetary gearbox by fusing vibration signal, sound signal, drive motor current, shaft speed, oil chip and other measurements, and then detected various system faults. Liu et al. [26] proposed a multi-sensor intelligent data fusion method based on the ant colony algorithm to realize fault detection of the gear box, aiming at the unreliability and instability of fault detection results of single sensor vibration signals. To avoid the disadvantage of combining a single information source with a decision method, a fault diagnosis method combining different types of sensor data sources and multiple classifier decisions is proposed in [27]. Banerjee et al. [28] proposed and investigated a hybrid method for fault signal classification based on sensor data fusion by using the support vector machine and short-term Fourier transform techniques. This method estimates the state of the target node by combining the information of surrounding nodes. This method has been successfully applied in individual formation [29], stability monitoring [30] and output coordination [31,32]. It can be concluded that distributed monitoring has better robustness and stability for single sensor monitoring in the sensor information fusion architecture. Considering multiple sensor information of the target monitoring system can improve the accuracy of state estimation more than considering single sensor information.

While the model-based method has been applied in fault diagnosis for high-speed train, there are still some problems. First, for the area monitored by multiple sensors, the estimation does not fully consider the influence of neighbor nodes which will further affect the later state estimation and fault diagnosis. Secondly, the statistical characteristics and the noise in the measurements collected in high-speed trains are normally unknown. The impacts of initial state and noise will be continuously amplified during the recursive estimation process, which may reduce the accuracy of fault diagnosis. Finally, the degradation of the system performance cannot be avoided. If the degradation is ignored, it will increase the false alarm rate of fault diagnosis and reduce the diagnostic accuracy. Aiming at solving the above problems, this paper proposes a distributed state estimation filter, which is combined with system state degradation characteristics to achieve fault diagnosis. This method is different from fault prognosis, the purpose of which is to analyze the possible faults in the future through the fault state feature [33], and then to provide guidance for future system health management. The purpose of this paper is to diagnose the existing faults of the degraded system. From the perspective of methods, the signal-based fault prognosis method [34] also has the constraint problem of data quantity and data type. The model-based fault prognosis method [35] mainly predicts the future fault generation time by analyzing the evolution trend of the fault. The method proposed in this paper is to diagnose the existing faults through the state evolution trend. They are similar, but still different.

Innovations and contributions of this article are as follows.

(1) Comprehensively consider the measuring point position and information acquisition method of a composite sensor. A distributed topology structure is established by taking the axle box bearing of an actual running gears system as an example. Based on this structure, a bilinear distributed filter is proposed, and the gain parameters of the filter are designed to minimize the mean square error.
(2) Unbiased constraint conditions are used to reduce the impact of the initial unknown information of nodes on state estimation. By constructing the difference to deal with the problem of colored noise in real measurements, estimation accuracy is improved.
(3) A nonlinear degradation model of the Wiener process considering temperature change characteristics is built to describe the state degradation phenomenon during train operation. The solution of nonlinear degradation process parameters is given by maximum likelihood estimation and combined with distributed filters to increase the accuracy of fault diagnosis.

Finally, to demonstrate the effectiveness of the proposed method, this paper conducts simulation experiments on the open dataset of rotating machinery bearings. The fault diagnosis results given are the same as actual experimental results. The proposed method was applied to operational monitoring data of a certain type of high-speed train running gears system, and fault diagnosis results obtained were consistent with a real situation.

This paper is arranged as follows. The second part describes the problem and introduces the preparation work. The third part proposes a method based on the Wiener process and multi-sensor filtering. The fourth part uses two case studies to test and analyze the above method. The final part concludes the article.

## 2. Preliminaries and Problem Formulations

**Figure 1:**
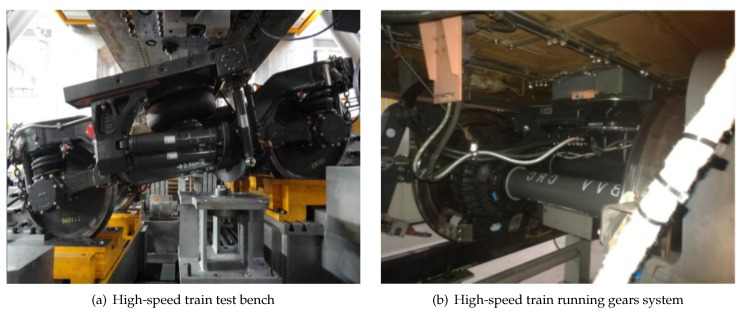
(**a**) High-speed train test bench; (**b**) high-speed train running gears system.

The running gears system is a complex mechanism, and its state information is an important indicator of traffic safety, as shown in Figure 1a,b. At present, the train fault diagnosis system is mainly composed of the train main engine, vehicle extension, preprocessing and composite sensors. The monitoring area of high-speed train operation consists of three or four sensors. Each sensor is integrated with two or more sensor units to detect different physical quantities and collect different types of data, such as temperature and vibration. Therefore, a single sensor is also called a composite sensor component. However, information acquisition in fault diagnosis based on single sensor information is not comprehensive or accurate. For the bearings of high-speed trains running a gears system, equipment status information from a single sensor is often significantly incomplete, which cannot provide an accurate and detailed data basis for subsequent fault diagnosis. Therefore, the fault diagnosis method based on single sensor information widely used in trains is limited in the accuracy of diagnosis and the completeness of information. To solve the problem, this paper abstracts a high-speed train running gears system into a multi-sensor system. The train control and management system processes the node information and gives full play to the advantages of a multi-node joint operation so that each node in a multi-sensor system can generate a consistent interpretation and description of the monitoring target. The reliability of sensor information is enhanced to achieve the purpose of collaborative monitoring and improve the accuracy of state estimation.

**Figure 2:**
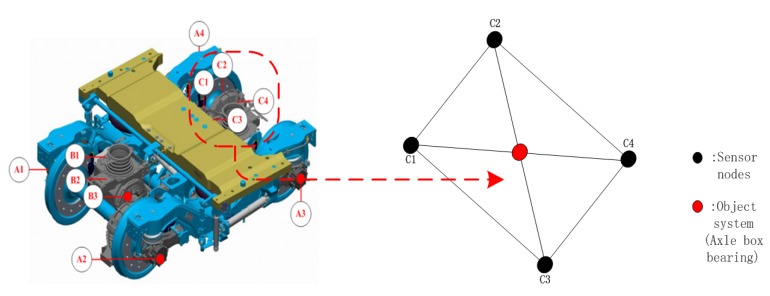
Distribution of measuring points of running gears system and topological structure of axle box bearing nodes.

Eleven composite sensors are installed in the support area of the monitoring bearing, and the installation direction needs to be consistent with the direction of the impact signal. The specific position is shown as follows, A1–A4: Measuring points of the axle box bearing; B1–B3: Measuring points for traction motor bearings; C1–C4: Measuring points for gearbox bearings. Taking the axle box bearing and its composite sensor in the above system as an example, a networked sensor system as shown in Figure 2 is established. The topology directed graph is represented by G=(V,E,D), where the vertex set is represented as V={1,2,3,4}, the set of edges is represented as ε⊆ν×ν.

This paper considers the effects of a sensor’s gain attenuation, colored measurement noise and accuracy reduction noise. The model of the target system is as follows.

(1) $$\begin{array}{c} x(k+1)=A(k)x(k)+B(k)u(k)+w(k)\\ y_{i}\left(k\right)=\lambda_{i}C_{i}\left(k\right)x\left(k\right)+D_{i}\left(k\right)u\left(k\right)+v_{i}\left(k\right)+s_{i}\left(k\right) \end{array}$$

To better conform to the changes in the actual state, the running characteristics of axle box bearings of the running section need to be analyzed. The axle box bearings of the running section are made of metal, and the specific heat capacity of metal is a function of temperature. In general, its specific heat capacity increases with increasing temperature. Therefore, the change of temperature of the axle box bearing will no longer conform to the law of linear change, and a bilinear term will be generated in a system equation. Consider describing the above system as the following bilinear form.

(2) $$\begin{array}{c} x(k+1)=A(k)x(k)+B(k)u(k)+N(k)u(k)x(k)+w(k)\\ y_{i}\left(k\right)=\lambda_{i}C_{i}\left(k\right)x\left(k\right)+D_{i}\left(k\right)u\left(k\right)+v_{i}\left(k\right)+s_{i}\left(k\right) \end{array}$$

where *k* is the discrete time index, u(k) is the control input signal, w(k) is the process noise and the mean is 0, the covariance is Q. v(k) represents measurement noise of the *i*-th sensor. s(k) is precision-degraded noise of the *i*-th sensor, the mean value is 0 and the covariance is O. λ is gain reduction of the *i*-th sensor. A(k),B(k),C(k) and D(k) are known coefficient matrices, and N(k)u(k)x(k) is a bilinear term.

In addition, as a key mechanical rotating part of the running gears system, the performance of the bearing plays an essential role in the safe running of the train. Generally speaking, mechanical equipment must go through a series of regular degradation stages from normal operation to complete failure. Therefore, taking the degradation process into account in the system model can more accurately describe the changing process of the actual system and increase the accuracy of state estimation. This paper considers the modeling of system state degradation based on the Wiener process. The more common nonlinear degradation model is as follows.

(3) $$\begin{array}{c} X\left(t\right)=X\left(0\right)+\int_{0}^{t}\;\mu (u;\theta )du+\sigma B(t) \end{array}$$

where μ(u;θ) is a non-linear function used to characterize the non-linear characteristics of the system degradation. θ is vector of unknown parameters contained in the function. σB(t) is the diffusion term used to describe the uncertainty of the degradation process. When the surface temperature of the friction pair rises to a certain extent for a short time, a secondary quenching layer and a high-temperature tempering layer will be produced on the surface of the bearing part. This burnt layer will cause obvious changes in the structure and performance of the surface of the bearing part, affecting the performance of the friction pair. This paper considers improving the above-mentioned Wiener process degradation model to achieve state degradation modeling.

## 3. Main Results and Discussion

**Figure 3:**
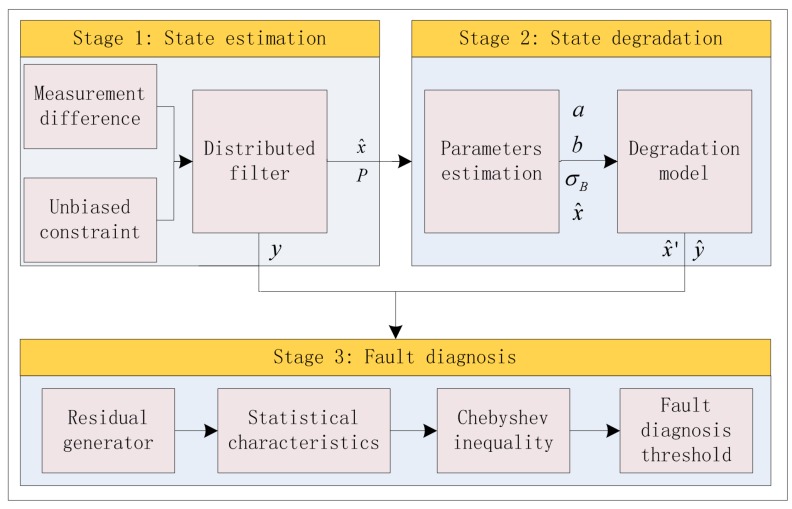
Fault diagnosis method framework of high-speed train running gears system.

The fault diagnosis method proposed in this paper is mainly divided into the following three stages: First, the distributed filtering design of multi-sensor systems. Second, model of the system state degradation based on the Wiener process. Third, fault diagnosis methods for the running gears system, shown in Figure 3.

### 3.1. Multi-Sensor Filter

Measurement noise during actual sensor operation is colored noise, the measurement noise in the differential form is constructed as follows.

(4) $$\begin{array}{c} v_{i}\left(k\right)=\psi_{i}\left(k-1\right)v_{i}\left(k-1\right)+\zeta_{i}\left(k-1\right) \end{array}$$

where ζ represents Gaussian white noise. To process *v*, an auxiliary signal *z* similar to the above is constructed.

(5) $$\begin{array}{cc} z_{i}\left(k\right) & =y_{i}\left(k\right)-\psi_{i}\left(k-1\right)v_{i}\left(k-1\right)\\ & =\lambda_{i}C_{i}\left(k\right)x\left(k\right)+D_{i}\left(k\right)u\left(k\right)+v_{i}\left(k\right)+s_{i}\left(k\right)-\psi_{i}\left(k-1\right)v_{i}\left(k-1\right)\\ & =\lambda_{i}C_{i}\left(k\right)x\left(k\right)+D_{i}\left(k\right)u\left(k\right)+\zeta_{i}\left(k-1\right)+s_{i}\left(k\right) \end{array}$$

The auxiliary signal *z* thus obtained will no longer contain colored noise terms, which is more convenient for processing. Continuing to use the equation of state of the original system, the measurement equation is described by auxiliary signal *z*, and the following equivalent equation of target system can be obtained:

(6) $$\begin{array}{c} x(k+1)=A(k)x(k)+B(k)u(k)+N(k)u(k)x(k)+w(k)\\ z_{i}\left(k\right)=\lambda_{i}C_{i}x\left(k\right)+D_{i}\left(k\right)u\left(k\right)+\zeta_{i}\left(k-1\right)+s_{i}\left(k\right) \end{array}$$

Considering the mutual influence between multi-sensor nodes, a distributed state estimation structure for bilinear systems is proposed as follows.

(7) $$\begin{array}{c} {\hat{x}}_{i}\left(k+1\right)=A\left(k\right){\hat{x}}_{i}\left(k\right)+L_{i}\left(k\right)u\left(k\right)+N\left(k\right)u\left(k\right){\hat{x}}_{i}\left(k\right)+\sum_{j\in Ni}H\left(k\right)[z_{j}\left(k\right)-\lambda_{i}C_{i}\left(k\right)x\left(k\right)] \end{array}$$

where ${\hat{x}}_{i}\left(k+1\right)$ represent the value of the estimated state of the *i*-th sensor, Ni is the set consisting of node *i* and its neighbors and H(k) is the filter gain of the multi-sensor.

In the process of state estimation, the uncertainty of the initial state will be transferred in the process of estimation iteration, leading to the deviation of state estimation. To satisfy the unbiasedness of the filter, the state estimation process is processed as follows, so that the mean of each state estimate is the same as the mean of the states, which is $E\left[\hat{x}\left(k\right)\right]=E\left[x\left(k\right)\right]$. The above formula is used as an unbiased constraint, so for the state estimation equation and the state equation at time k+1:

(8) $$\begin{array}{c} E\left[{\hat{x}}_{i}\left(k+1\right)\right]=A\left(k\right)E\left[{\hat{x}}_{i}\left(k\right)\right]+L_{i}\left(k\right)u\left(k\right)+N\left(k\right)u\left(k\right)E\left[{\hat{x}}_{i}\left(k\right)\right]+\sum_{j\in Ni}H\left(k\right)D_{i}\left(k\right)u\left(k\right)\\ E\left[x_{i}\left(k+1\right)\right]=A\left(k\right)E\left[x_{i}\left(k\right)\right]+B_{i}\left(k\right)u\left(k\right)+N\left(k\right)u\left(k\right)E\left[x_{i}\left(k\right)\right]\\ E\left[{\hat{x}}_{i}\left(k+1\right)\right]-E\left[x_{i}\left(k+1\right)\right]=\left[L_{i}\left(k\right)-B_{i}\left(k\right)+\sum_{j\in Ni}H(k)D_{i}\left(k\right)\right]u\left(k\right)=0 \end{array}$$

Therefore, the unbiased constraint can be equivalent to:

(9) $$\begin{array}{c} L_{i}\left(k\right)-B_{i}\left(k\right)+\sum_{j\in Ni}H\left(k\right)D_{i}\left(k\right)=0 \end{array}$$

Next, the gain parameter *H* of the filter is obtained by minimizing the trace of estimated error covariance. The estimated error function is established as follows.

(10) $$\begin{array}{c} e_{i}\left(k+1\right)=\left[{\hat{x}}_{i}\left(k+1\right)-x_{i}\left(k+1\right)\right] \end{array}$$

The gain parameter *H* of the filter is expressed as:

(11) $$\begin{array}{cc} H_{B} & =\underset{H}{\arg\min}E\left\{e^{T}\left(k+1\right)\cdot e\left(k+1\right)\right\}\\ & =\underset{H}{\arg\min}E\left\{trace\left[e\left(k+1\right)\cdot e^{T}\left(k+1\right)\right]\right\}\\ & =\underset{H}{\arg\min}E\left\{trace\left[\left(\sum_{j\in Ni}H\left(k\right)\zeta_{j}\left(k-1\right)+H\left(k\right)S_{j}\left(k\right)-w_{j}\left(k\right)\right)\cdot {\left(\cdots \right)}^{T}\right]\right\}\\ & =\underset{H}{\arg\min}trace\left\{\sum_{j\in Ni}[H\left(k\right)R_{j}\left(k-1\right)H^{T}\left(k\right)+H\left(k\right)O_{j}\left(k\right)H^{T}\left(k\right)-Q_{j}\left(k\right)\right]\} \end{array}$$

Using the unbiased constraint as the condition for minimizing the above formula, the Lagrange function is constructed.

(12) $$\begin{array}{cc} Js(H,\lambda )= & \left[\sum_{j\in N_{i}}H\left(k\right)R_{j}\left(k-1\right)H^{T}\left(k\right)+H\left(k\right)O_{j}\left(k\right)H^{T}\left(k\right)-Q_{i}\left(k\right)\right]\\ & +\lambda [L_{i}\left(k\right)-B_{i}\left(k\right)+\sum_{j\in Ni}H\left(k\right)D_{j}\left(k\right)] \end{array}$$

Then, *H* can be expressed as:

(13) $$\left\{\begin{array}{c} H\left(k\right)=\sum_{j\in N_{i}}-\frac{1}{2}\lambda {\left[R_{j}\left(k-1\right)+O_{j}\left(k\right)\right]}^{-1}\cdot D_{j}\left(k\right)\\ \sum_{j\in N_{i}}H\left(k\right)D_{j}\left(k\right)=B_{i}\left(k\right)-L_{i}\left(k\right) \end{array}\right.$$

In summary, the analytical solution of the gain parameter *H* of the distributed filter is finally obtained as:

(14) $$\begin{array}{c} H\left(k\right)=\left[B_{i}\left(k\right)-L_{i}\left(k\right)\right]\sum_{j\in N_{i}}{\left\{{\left[R_{j}\left(k-1\right)+O_{j}\left(k\right)\right]}^{-1}D_{j}\left(k\right)\right\}}^{-1}D_{j}^{-1}\left(k\right){\left[R_{j}\left(k-1\right)+O_{j}\left(k\right)\right]}^{-1}D_{j}\left(k\right) \end{array}$$

**Remark** **1.** *This paper only considers the measurement noise of a system as colored noise, instead of colored process noise. The reason is that the state degradation process is the main reason that directly affects system change. The characteristics of process noise have little effect on the system. If process noise is processed in the same form, it will increase the computational complexity.*

### 3.2. Parameter Estimation of State Degradation

Considering the effects of random shocks and environmental factors (temperature), this paper builds a nonlinear degradation process based on the Wiener degradation model and provides a solution for parameter estimation.

Construct a nonlinear state degradation model.

(15) $$\begin{array}{c} X\left(t\right)=X\left(0\right)+\int_{0}^{t}\;\mu \left(u;\theta \right)du+\sum_{i=1}^{N}J_{i}+u\left(t\right)x_{state}\left(t\right)+\sigma B\left(t\right) \end{array}$$

where X(t) is the degradation process driven by the standard drift Brownian motion B(t), μ(u;θ) is a non-linear function, *J* is the amplitude of random shocks, *N* represent the random shock, and u(t)x(t) is the environmental factor on the system state degraded parameters. It can be seen that when μ(u;θ)=μ, the degradation process becomes a linear degradation model. In order to describe the above models in detail, this paper gives a parameter estimation method for a class of nonlinear models.

(16) $$\begin{array}{c} \mu \left(t;\theta \right)=abt^{b-1}\;\;,\;X\left(t\right)=X\left(0\right)+at^{b}+\sum_{i=1}^{N}J_{i}+u\left(t\right)x_{state}\left(t\right)+\sigma B\left(t\right) \end{array}$$

The unknown parameters of the drift and diffusion terms in the degradation process are estimated, assuming current time is $t_{k}$, and the historical detection data of equipment degradation is $x_{1},x_{2},\cdots ,x_{k}$, where *x* is obtained by sampling the degradation process at equal intervals. ${\tilde{X}}_{1:k}$ represents the incremental degradation process.

**Theorem** **1.** *The average value of increment ${\tilde{X}}_{1:k}$ of the above degradation process is:*

(17) $$\begin{array}{cc} \; & aE{\left[t_{1}^{b}-t_{0}^{b}\;\;\;t_{2}^{b}-t_{1}^{b}\;\;\;\cdots \;\;\;t_{i+1}^{b}-t_{i}^{b}\right]}^{T}\\ \; & -E{\left[u_{1}{x_{state}}_{1}-u_{0}{x_{state}}_{0}\;\;\;u_{2}{x_{state}}_{2}-u_{1}{x_{state}}_{1}\;\;\;\cdots \;\;\;u_{i+1}{x_{state}}_{i+1}-u_{i+1}{x_{state}}_{i+1}\right]}^{T} \end{array}$$

*The variance is $\sigma_{B}^{2}\cdot Q$ and follows a normal distribution.*

**Proof of Theorem 1.**  Tectonic degradation increment:

(18) $$\begin{array}{cc} X(i+1) & =x(i+1)-x(i)\\ & =a{\left[t\left(i+1\right)\right]}^{b}+u\left[t\left(i+1\right)\right]x_{state}\left[t\left(i+1\right)\right]+\sigma_{B}B\left[t\left(i+1\right)\right]\\ & -at{\left(i\right)}^{b}-u\left[t\left(i\right)\right]x_{state}\left[t\left(i\right)\right]-\sigma_{B}B\left[t\left(i\right)\right]\\ & =a\left[t{\left(i+1\right)}^{b}-t{\left(i\right)}^{b}\right]+\Delta +\sigma_{B}\left\{B\left[t\left(i+1\right)\right]-B\left[t\left(i\right)\right]\right\} \end{array}$$

where Δ can represent the influence of the environment (temperature) on state degradation, and the mean value of the above equation can be obtained:

(19) $$\begin{array}{c} E\left[X\left(i+1\right)\right]=aE\left[t{\left(i+1\right)}^{b}-t{\left(i\right)}^{b}\right]-E\left[\Delta \right] \end{array}$$

The covariance can be expressed as:

(20) $$\begin{array}{cc} & \mathrm{var}[X(i+1)\cdot X(j+1)]\\ = & \mathrm{var}\{x[t(i+1)]-x[t(i)]\}\cdot \{x[t(j+1)]-x[t(j)]\}\\ = & E\left\{x\left[t\left(i+1\right)\right]-x\left[t\left(i\right)\right]\right\}\left\{x\left[t\left(j+1\right)\right]-x\left[t\left(j\right)\right]\right\}-E\left\{x\left[t\left(i+1\right)\right]-x\left[t\left(i\right)\right]\right\}\cdot E\left\{x\left[t\left(j+1\right)\right]-x\left[t\left(j\right)\right]\right\}+\sigma_{B}\left(\cdot \right)\\ = & E\left\{a^{2}\left\{{\left[t\left(i+1\right)\right]}^{b}-t{\left(i\right)}^{b}\right\}\left[t{\left(j+1\right)}^{b}-t{\left(j\right)}^{b}\right]\right\}+E\left\{a\left[t{\left(i+1\right)}^{b}-t{\left(i\right)}^{b}\right]\cdot \Delta \left(j\right)\right\}\\ + & E\left\{\Delta \left(i\right)\cdot a\left[t{\left(j+1\right)}^{b}-t{\left(j\right)}^{b}\right]\right\}+E\left[\Delta \left(i\right)\Delta \left(j\right)\right]-E\left\{a^{2}\left[t{\left(i+1\right)}^{b}-t{\left(i\right)}^{b}\right]\left[t{\left(j+1\right)}^{b}-t{\left(j\right)}^{b}\right]\right\}\\ - & aE\left[t{\left(i+1\right)}^{b}-t{\left(i\right)}^{b}\right]\Delta \left(i\right)-\Delta \left(j\right)\cdot aE\left[t{\left(j+1\right)}^{b}-t{\left(j\right)}^{b}\right]+\sigma_{B}\left(\cdot \right)\\ = & E\{\sigma_{B}\left\{B\left[t\left(i+1\right)\right]-B\left[t\left(i\right)\right]\right\}\left\{a\left[t{\left(j+1\right)}^{b}-t{\left(j\right)}^{b}\right]+\Delta \left(j\right)\right\}\}\\ + & E\{\sigma_{B}\left\{B\left[t\left(j+1\right)\right]-B\left[t\left(j\right)\right]\right\}\left\{a\left[t{\left(i+1\right)}^{b}-t{\left(i\right)}^{b}\right]+\Delta \left(i\right)\right\}\}\\ + & E\{\sigma_{B}^{2}\left\{B\left[t\left(i+1\right)\right]-B\left[t\left(i\right)\right]\right\}\cdot \left\{B\left[t\left(j+1\right)\right]-B\left[t\left(j\right)\right]\right\}\}\\ = & \sigma_{B}^{2}E\left\{\left\{B\left[t\left(i+1\right)\right]-B\left[t\left(i\right)\right]\right\}\cdot \left\{B\left[t\left(j+1\right)\right]-B\left[t\left(j\right)\right]\right\}\right\} \end{array}$$

where $\sigma_{B}\left(\cdot \right)$ represents the term associated with $\sigma_{B}$.

Set Q=E{{B[t(i+1)]−B[t(i)]}{B[t(j+1)]−B[t(j)]}}, the variance of the degenerate increment is $\sigma_{B}^{2}\cdot Q$.

Construct the likelihood function:

(21) $$\begin{array}{c} l\left(\theta ;\sigma_{B}|{\tilde{X}}_{0:k}\right)=\frac{1}{\sqrt{2\pi }\cdot \sigma_{B}\sqrt{Q}}\cdot e^{-\frac{{\left({\tilde{X}}_{1:k}-a\mu_{1:k}+\Delta_{1:k}\right)}^{2}}{2\sigma_{B}^{2}Q}} \end{array}$$

according to the above likelihood function, the desired *a* and $\sigma_{B}$ can be obtained:

(22) $$\begin{array}{c} \frac{d\ln(l)}{da}=\frac{{\tilde{X}}_{1:k}\cdot \mu_{1:k}+\Delta_{1:k}\cdot \mu_{1:k}}{2\sigma_{B}^{2}Q}-\frac{\mu_{1:k}^{2}\cdot a}{\sigma_{B}^{2}Q}=0\;\;;\;\;\hat{a}=\frac{{\tilde{X}}_{1:k}+\Delta_{1:k}}{2\mu_{1:k}} \end{array}$$

(23) $$\begin{array}{c} \frac{d\ln(l)}{d\sigma_{B}}=-\frac{1}{\sigma_{B}}+\frac{{\left({\tilde{X}}_{1:k}-a\mu_{1:k}+\Delta_{1:k}\right)}^{2}}{\sigma_{B}^{3}Q}=0\;\;;\;\;{\hat{\sigma }}_{B}=\frac{{\tilde{X}}_{1:k}-a\mu_{1:k}+\Delta_{1:k}}{\sqrt{Q}} \end{array}$$

The estimated values of *a* and $\sigma_{B}$ are substituted into the log-likelihood function, and the log-likelihood function is maximized by the simplex method to get *b*. □

**Remark** **2.** *The state degradation model needs to be combined with the state estimation model. The continuous function of the degradation process needs to be discretized.*

### 3.3. Residual Generator Design

Discretizing the state variables in stage 2 and combining them with the state variables in stage 1:

(24) $$\begin{array}{c} x^{\prime }\left(k\right)=x\left(k\right)+X\left(k\right)\\ {\hat{x}}^{\prime }\left(k\right)=\hat{x}\left(k\right)+X\left(k\right) \end{array}$$

Constructing a residual generator:

(25) $$\begin{array}{c} T_{i}\left(k\right)=y_{i}\left(k\right)-{\hat{y}}_{i}\left(k\right) \end{array}$$

where ${\hat{y}}_{i}\left(k\right)=\lambda_{i}C_{i}\left(k\right)\hat{x}\left(k\right)+D_{i}\left(k\right)u\left(k\right)$, and then:

(26) $$\begin{array}{cc} T_{i}\left(k\right) & =\lambda_{i}C_{i}\left(k\right)\left[x\left(k\right)-\hat{x}\left(k\right)\right]+v_{i}\left(k\right)+s_{i}\left(k\right)\\ & =\lambda_{i}C_{i}\left(k\right)\cdot E^{+}\left(k-1\right)E\left(k-1\right)\cdot \left[x\left(k\right)-\hat{x}\left(k\right)\right]+v_{i}\left(k\right)+s_{i}\left(k\right)\\ & =\lambda_{i}C_{i}\left(k\right)\cdot E^{+}\left(k-1\right)\{A\left(k-1\right)x\left(k-1\right)+N\left(k-1\right)u\left(k-1\right)x\left(k-1\right)+B\left(k-1\right)u\left(k-1\right)+w\left(k-1\right)\\ & -[A\left(k-1\right){\hat{x}}_{i}\left(k-1\right)+L_{i}\left(k-1\right)u\left(k-1\right)+N\left(k-1\right)u\left(k-1\right){\hat{x}}_{i}\left(k-1\right)\\ & +\sum_{j\in Ni}H\left(k-1\right)[Z_{j}\left(k-1\right)-\lambda_{j}C_{j}\left(k-1\right)x\left(k-1\right)]]\}+v_{i}\left(k\right)+s_{i}\left(k\right)\\ & =\lambda_{i}C_{i}\left(k\right)\cdot E^{+}\left(k-1\right)\{A\left(k-1\right)\left[x_{i}\left(k-1\right)-{\hat{x}}_{i}\left(k-1\right)\right]+N\left(k-1\right)u\left(k-1\right)\left[x_{i}\left(k-1\right)-{\hat{x}}_{i}\left(k-1\right)\right]\\ & +w\left(k-1\right)-\sum_{j\in Ni}H\left(k-1\right)[\zeta_{j}\left(k-2\right)+s_{j}\left(k-1\right)]\}+v_{i}\left(k\right)+s_{i}\left(k\right) \end{array}$$

**Theorem** **2.** *The given residual generator satisfies the following distribution*

(27) $$\begin{array}{c} Ti\left(k\right)\{E\left[v_{i}\left(k\right)\right],\;\;\tilde{P}\left(k\right)\} \end{array}$$

*where the mean value and variance of v are derived by following equation:*

(28) $$\begin{array}{c} v_{i}\left(k\right)=y_{i}\left(k\right)-\lambda_{i}C_{i}\left(k\right){\hat{x}}_{i}\left(k\right)-D_{i}\left(k\right)u\left(k\right)-s_{i}\left(k\right) \end{array}$$

**Proof of Theorem 2.**  (29) $$\begin{array}{c} E\left[Ti\left(k\right)\right]=E\left[v_{i}\left(k\right)\right] \end{array}$$

(30) $$\begin{array}{cc} \tilde{P}\left(k\right) & =E[Ti\left(k\right)\cdot Ti^{T}\left(k\right)]\\ & =\left[\lambda_{i}C_{i}\left(k\right)\right]\cdot E^{+}\left(k-1\right)\left\{Q\left(k-1\right)-\sum_{j\in Ni}H\left(k-1\right)\left[R\left(k-2\right)+O\left(k-1\right)\right]H^{T}\left(k-1\right)\right\}\\ & \cdot {\left\{\left[\lambda_{i}C_{i}\left(k\right)\right]\cdot E^{+}\left(k-1\right)\right\}}^{T}+E\left[v_{i}\left(k\right)\cdot {v_{i}}^{T}\left(k\right)\right]+O\left(k\right) \end{array}$$

The fault diagnosis threshold is obtained by the Chebyshev inequality:

(31) $$\begin{array}{c} \mathrm{Pr}\left(\mu \left(X\right)-\alpha \sigma \left(X\right)\leq X\leq \mu \left(X\right)+\alpha \sigma \left(X\right)\right)\geq 1-\frac{1}{\alpha^{2}} \end{array}$$

□

## 4. Practical Verifications and Discussion

### 4.1. State Estimation

**Figure 4:**
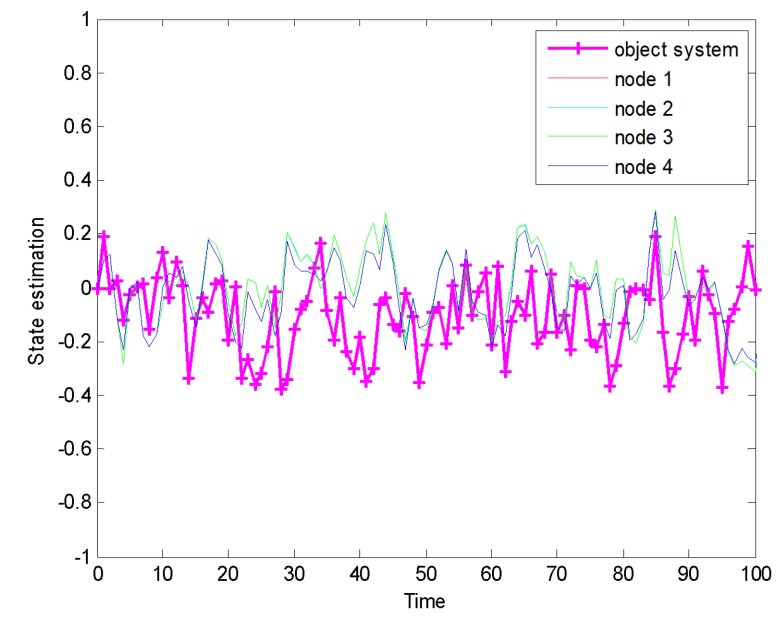
Distributed state estimation.

Firstly, this paper presents the state estimation curve of four nodes in monitoring systems, as shown in Figure 4.

**Figure 5:**
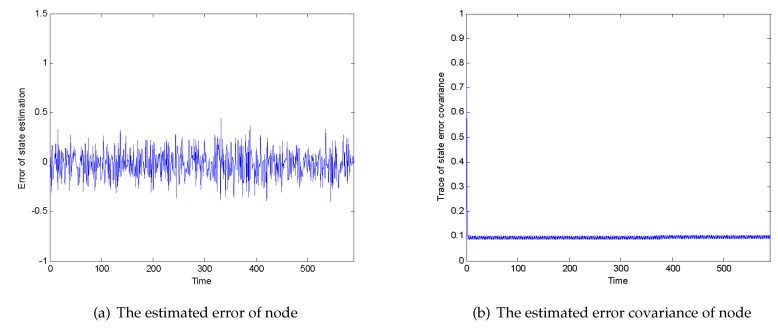
(**a**) The estimated error of a node; (**b**) the estimated error covariance of a node.

Figure 5a,b, respectively, shows the state estimation error value and state estimation error covariance of a single node. It can be seen that the state estimation error keeps within ±0.5, while the state estimation error covariance converges to a smaller value, proving the effectiveness of the filter.

### 4.2. Cincinnati Dataset

First, the 2003 Cincinnati test 1 bearing 2 channel 4 data was used for simulation experiments. The sampling time of this dataset is from 12:06:24 on 22 October 2003 to 23:39:56 on 25 November 2003. The test bench device contains four bearings, and the AC motor is coupled to the shaft through a friction band. The speed was kept at 2000 RPM. A 6000-pound radial load was applied to the shaft and bearings. Each dataset consists of a single file of 1 second. The sampling rate is 20 kHz, and 20,480 points are saved at a time.

**Figure 6:**
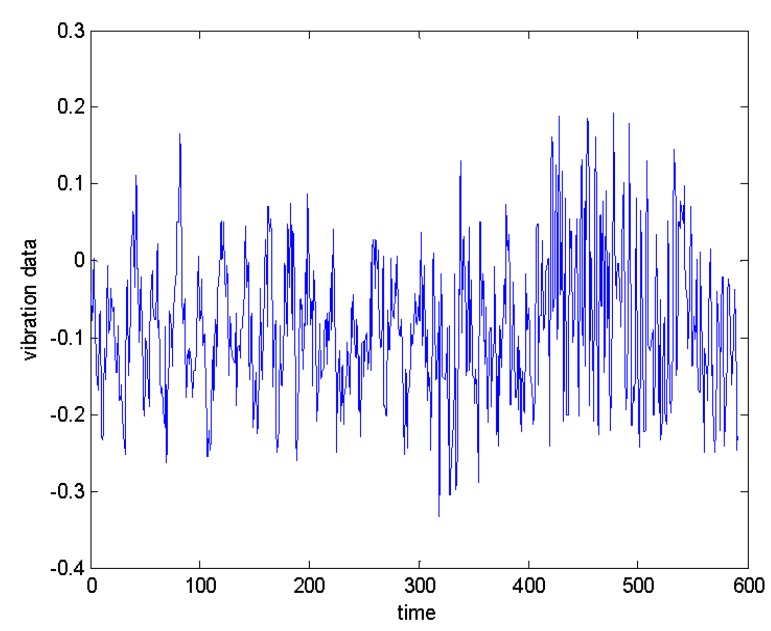
Cincinnati bearing vibration data values.

The degree of bearing damage in the experiment is related to operating time of device, and the corresponding data format will also change. Generally speaking, it can be clearly seen from the amplitude of the data whether the monitoring system has failed. Therefore, to better illustrate the reliability of algorithm, this article selects some data from above data set, including non-faulty data points, early fault data points, and severe fault data points. In this paper, the initial wear state of the bearing is defined as early failure, but it does not affect the continued operation of the whole system. A serious fault may refer to the state of near failure of the bearing in the period before the end of the experiment. At this point, the serious fault has affected the operation of system and even caused some damage to bearing itself. Therefore, it is of greater practical significance to the diagnosis of serious fault, and diagnosis of early fault has certain guiding significance to prediction and maintenance of the future.To better demonstrate effectiveness of the proposed method, data with small amplitude differences were selected from above three types of data for verification, as shown in Figure 6.

**Figure 7:**
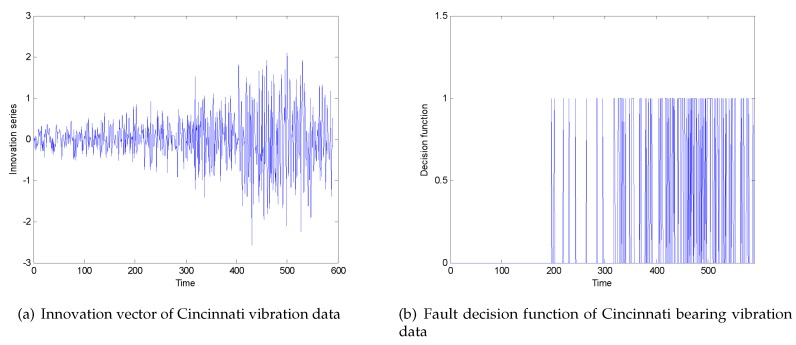
(**a**) Innovation vector of Cincinnati vibration data; (**b**) fault decision function of Cincinnati bearing vibration data.

Among them, time 0–200 is a non-fault data point; time 200–400 is an early fault data point; 400–600 is a severe fault data point. After passing the filter with state degradation, the innovation vector is obtained as shown in Figure 7a. Taking the adjustable parameter α=7 in the Chebyshev inequality, the threshold value obtained are ±0.53. For the convenience of viewing, a decision function as shown in Figure 7b is given. The threshold exceeded is set to 1, and the threshold not exceeded is set to 0. It can be seen that the fault occurred from 200–400 moments, but because the fault occurred early, the number is relatively sparse, and the fault is a serious fault at 400–600 moments, and the number of faults is relatively large.

### 4.3. High-Speed Train Temperature Dataset

**Figure 8:**
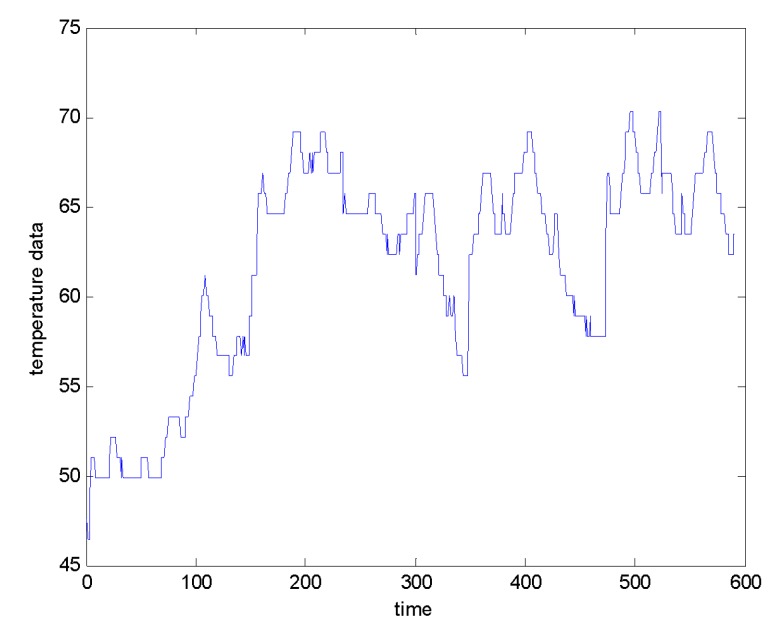
Temperature data of running gears system.

This section uses an actual running gears system to prove the effectiveness of proposed algorithm. Selecting data of the failed train and the data of non-faulted train. The data is derived from temperature value of temperature sensor on the right position of the 2 axis of one-run part of the train, in Figure 8.

**Figure 9:**
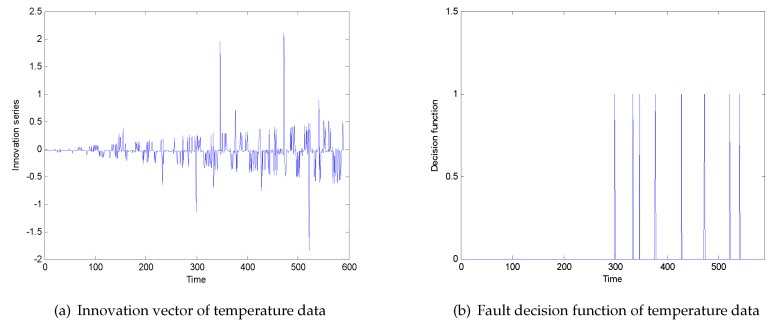
(**a**) Innovation vector of temperature data; (**b**) fault decision function of temperature data.

Time 0–300 is a non-faulty data point, and time 300–600 is a faulty data point. After passing through the filter with state degradation, the innovation vector is obtained as shown in Figure 9a. Taking the adjustable parameter α=7 of Chebyshev inequality, the obtained threshold is ±0.56. Similarly, for the convenience of viewing, the decision function shown in Figure 9b is given, and it can be seen that there is a fault in time 300–600, which is consistent with the error information given by actual high-speed train.

### 4.4. Performance Comparison

In this paper, the proposed algorithm is compared with algorithms commonly used in fault diagnosis. Partial data of the Cincinnati public dataset is selected for fault diagnosis. The diagnostic accuracy is shown in following table (see Table 1). Among them, FNR is the rate of false negatives and FPR represents the rate of false positives.

## 5. Conclusions

The stable operation of high-speed trains is closely related to the running gears system. This paper proposes a method based on the Wiener state degradation process and multi-sensor filtering for running gears system diagnosis. First, a distributed topology diagram of running gears system axle box bearings and its composite sensors is established by analyzing a running system of a high-speed train. Secondly, a distributed filter is proposed to the problem of unknown initial information of sensor nodes by the unbiased constraint condition, considering the problems of gain attenuation and precision decline, and the gain parameters of the filter are calculated under the premise of minimum error mean square error. Then, to improve the fault diagnosis accuracy of the running gears system, this paper built a nonlinear degradation model of the Wiener process considering the factor of temperature, and estimated parameters through maximum likelihood estimation. Finally, a fault diagnosis threshold is obtained by using the Chebyshev inequality. The performance of the method is verified by an open dataset of rotating mechanical bearings and temperature data of a certain type of high-speed rail running gears system. The result is as expected.

The method proposed in this paper is more meaningful for practical engineering, and improves the accuracy of fault diagnosis for systems that cannot obtain available data. This method aims to diagnose early faults. For other types of dynamic systems, thresholds can be set based on their actual characteristics and the definition of the corresponding fault level. Future research work starts from the correlation between states, decouples filter states, and considers a non-linear degradation problem of non-Markov processes, making the model closer to the actual process. 

## Figures and Tables

**Table 1:** Performance comparison.

Methods	FNR	FPR
State-degraded distributed filter	0.26%	0.52%
Kalman filter	1.28%	6.03%
Extended Kalman filter	2.04%	5.46%
